# Data of chemical composition of the particles from OH-initiated oxidation of 1,3,5-trimethylbenzene

**DOI:** 10.1016/j.dib.2022.108152

**Published:** 2022-04-11

**Authors:** Xiaoxiao Lin, Xiaofeng Tang, Zuoying Wen, Bo Long, Christa Fittschen, Xuejun Gu, Yang Zhang, Weijun Zhang

**Affiliations:** a Laboratory of Atmospheric Physico-Chemistry, Anhui Institute of Optics and Fine Mechanics, HFIPS, Chinese Academy of Sciences, Hefei, Anhui 230031, China; b School of Materials Science and Engineering, Guizhou Minzu University, Guiyang, Guizhou 550025, China; c University Lille, CNRS, UMR 8522, PC2A – Physicochimie des Processus de Combustion et de l'Atmosphère, Lille F-59000, France

**Keywords:** Particle-phase product, 1,3,5-trimethylbenzene, Secondary organic aerosol, Molecular structures, VUV photoionization aerosol mass spectrometry

## Abstract

This paper presents the data of chemical composition of the particles from OH oxidation reaction of 1,3,5-trimethylbenzene (1,3,5-TMB). The particle-phase compositions are measured on-line by using a vacuum ultraviolet (VUV) photoionization aerosol mass spectrometer. The assignments of the major peaks of photoionization mass spectrum, as well as their molecular structures, are presented. The optimized structures of the reactants, intermediates and transition states involved in the reaction of the bicyclic peroxy radical with HO_2_ are shown. The reaction routes of the OH-initiated oxidation of the deuterated 1,3,5-TMB sample are also calculated and displayed for comparison. The data presented here is related to the paper “Direct observation of the particle-phase bicyclic products from OH-initiated oxidation of 1,3,5-trimethylbenzene under NOx-free conditions” by Lin et al. (2022).

## Specifications Table

**Table:** 

Subject	Chemistry
Specific subject area	Atmospheric chemistry
Type of data	Table Figure Graph
How the data were acquired	Mass spectra: Vacuum ultraviolet (VUV) photoionization aerosol mass spectrometer. Structures and reaction routes: ChemBioDraw Ultra 12.0, Gaussian 16 and GaussView 6.
Data format	Raw Analysed Filtered
Description of data collection	The mass spectra of the particle-phase products from OH oxidation reaction of 1,3,5-TMB were measured by using the VUV photoionization aerosol mass spectrometer. The molecular structures were drawed manually by using Chemdraw ultra 12.0. The geometric structures were optimized by using Gaussian 16 program. The GaussView as the graphical interface for Gaussian 16 was used to open the output files.
Data source location	Institution: Anhui Institute of Optics and Fine Mechanics, CAS City/Town/Region: Hefei, Jinggang, 230031 Country: China
Data accessibility	Data are available with this article. The data are also available via ProteomeXchange with the dataset identifier PXD030839. https://www.ebi.ac.uk/pride/archive/projects/PXD030839
Related research article	X. Lin, X. Tang, Z. Wen et al., Direct observation of the particle-phase bicyclic products from OH-initiated oxidation of 1,3,5-trimethylbenzene under NOx-free conditions, Atmospheric Environment. (2022, 271, 118914). 10.1016/j.atmosenv.2021.118914

## Value of the Data

• The data presented in this paper are valuable for the characterization of secondary organic aerosol (SOA) from oxidation of aromatic compounds.
• The data can help researchers to better understand the reaction mechanisms of the bicyclic peroxy radical involved in the atmospheric oxidation.
• The data would serve as a reference for studying or analysing the chemical compositions of SOA from OH-initiated oxidation of 1,3,5-TMB.

## 1 Data Description

The data of this paper includes one table, two figures and the mass spectrometry raw data open to readers via ProteomeXchange. Concretely, Table 1 lists the molecular structures of the major particle-phase products from OH-initiated oxidation of 1,3,5-trimethylbenzene (1,3,5-TMB). It shows the mass (m/z) and the name of these products and their corresponding molecular structures which were drawn with the ChemBioDraw Ultra program. The oxygen-containing functional groups in these molecular structures are presented in red.

**Table 1:** The assignments of the particle-phase products from OH-initiated oxidation of 1,3,5-TMB.

m/z	Product	Structure	m/z	Product	Structure
58	acetone		130	citraconic acid	
60	acetic acid			mesaconic acid	
72	methyl glyoxal		134	malic acid	
74	hydroxyacetone			2-hydroxy-3-oxobutaneperoxoic acid	
	oxo-acetic acid			3,5-dimethyl benzaldehyde	
84	butenedial		136	2,4,6-trimethyl phenol	
86	oxo-propanedial		138	3,5-dimethyl-phenyl-hydroperoxide	
100	2,3-dioxobutanal		144	2-hydroxy-2-methyl-3,4-dioxo-pentanal	
	butanoic acid		150	3,5-dimethylbenzoic acid	
102	2-hydroxy-2-methyl-malonaldhyde		152	(3,5-dimethyl-phenyl)-methyl-hydroperoxide	
110	3-methyl-5-methylidene-5-(2H)furanone		154	4-methyl-hept-4-ene-2,3,6-trione	
112	2-methyl-4-oxo-2-pentenal		156	5-hydroxy-4,6-dioxo-2-heptenal	
	3,5-dimethyl-2(3H)-furanone		166	3,5-dimethyl-benzenecarboperoxoic acid	
	3-methyl-2,5-furandione		168	2-methyl-3-(1-methyl-3-oxobut-1-enyl)-oxirane-2-carbaldehyde	
114	pentane-2,3,4-trione		184	8-hydroxy-1,3,5-trimethyl-6,7-dioxa-bicyclo[3.2.1]oct-3-en-2-one	
116	3-hydroxy-pentane-2,4-dione		186	1,3,5-trimethyl-6,7-dioxa- bicyclo[3.2.1] oct-3-ene-2,8-diol	
118	2-hydroperoxy-2-methyl-malonaldehyde		202	8-hydroxy-1,3,5-trimethyl-6,7-dioxa-bicyclo[3.2.1]oct-3-en-2- hydroperoxide	
128	2-methyl-4-oxo-pent-2-enoic acid		218	8-hydroxy-1,3,5-trimethyl-6,7-dioxa-bicyclo[3.2.1]oct-3-en-2- trioxide	

**Fig. 1:**
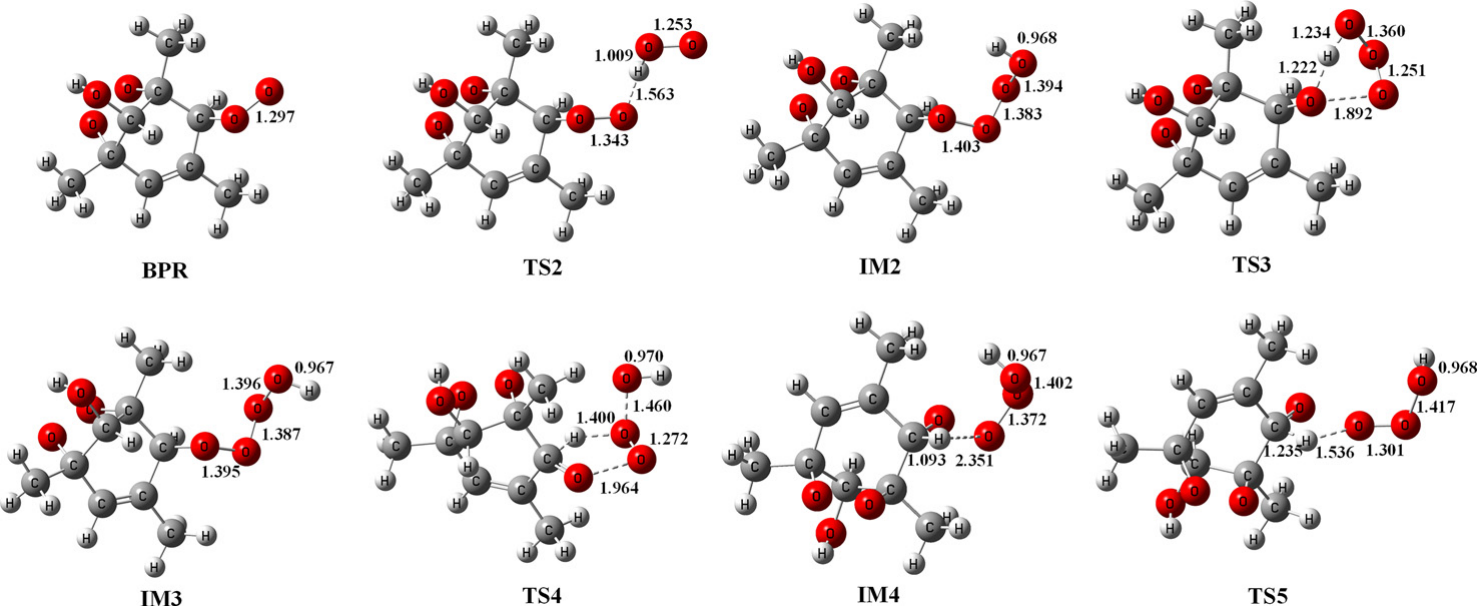
Geometric structures of the reactants, intermediates and transition states of the reaction between the O_2_-bridged bicyclic peroxy radical (BPR) and HO_2_ computed at the M06-2X/MG3S level of theory on the singlet potential energy surfaces.

Fig. 1 presents the selected geometric structures of the reactants, intermediates and transition states of the reaction between the O_2_-bridged bicyclic peroxy radical (BPR) and HO_2_ computed at the M06-2X/MG3S level of theory on the singlet potential energy surfaces. The geometric structures were optimized by using the Gaussian 16 program. In Fig. 1, BPR is the abbreviation of the bicyclic peroxy radical, the abbreviation of TS stands for the trasition states, and IM stands for intermediates. H atoms, C atoms and O atoms are colored in white, grey and red, respectively. The selected bond distances have been given in angstroms.

**Fig. 2:**
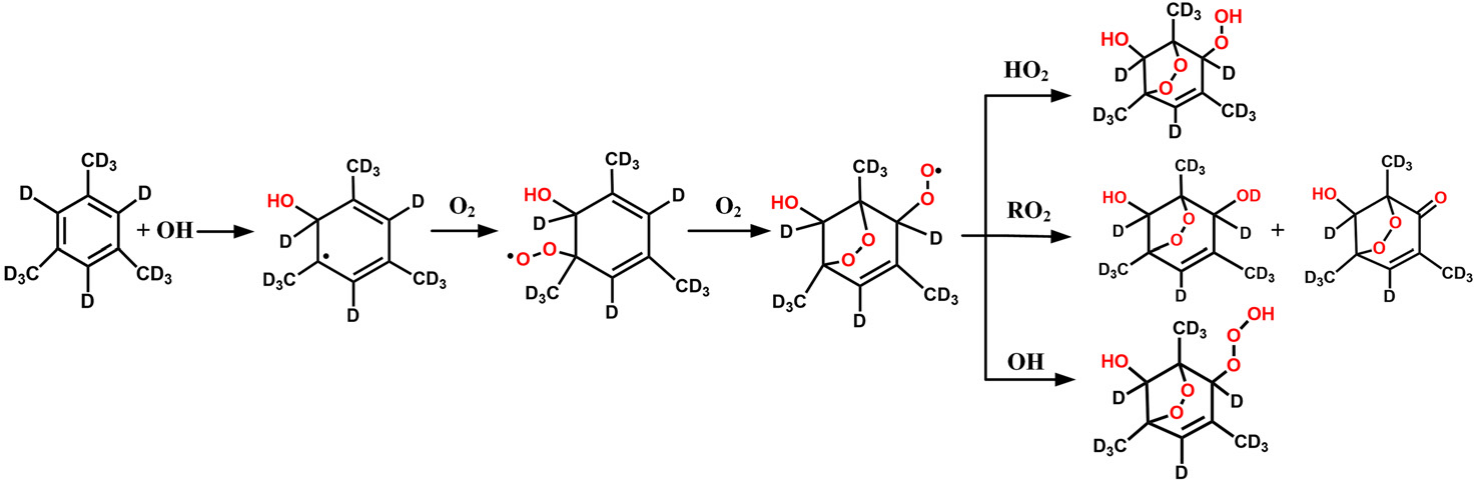
The reaction routes of the deuterated 1,3,5-TMB (C_9_D_12_) with OH radical under NO*x* free conditions.

Fig. 2 presents the reaction routes of the OH initiated oxidation of the deuterated 1,3,5-TMB (C_9_D_12_) under NOx free conditions. The molecular structures were drawn with the ChemBioDraw Ultra program. The detailed description of the reaction routes can be seen in the Section 2.6 below.

In addition, Proteome Xchange with the dataset identifier PXD030839 lists the mass spectrometry raw data file (raw data.zip) which can be downloaded publicly. The first two columns of data present the mass (m/z) and the intensity (a.u.) of the mass spectrum from the OH-initiated oxidation of 1,3,5-TMB, and the last two columns show the mass (m/z) and the intensity (a.u.) of the mass spectrum from the OH-initiated oxidation of deuterated 1,3,5-TMB (C_9_D_12_). The corresponding mass spectrum figure file (MS.jpg) can also be seen on-line via the link https://www.ebi.ac.uk/pride/archive/projects/PXD030839.

## 2 Experimental Design, Materials and Methods

### 2.1 Materials

1,3,5-TMB (98%) was purchased from Alfa Aesar without further purification, the deuterated 1,3,5-TMB-d12 (98 atom% D) was purchased from Sigma–Aldrich (Saint Louis, MO, USA), and H_2_O_2_ (35% in H_2_O) was obtained from Acros Organics.

### 2.2 Experimental design

A simulation chamber made of a Teflon bag and surrounded by six ultraviolet lamps (Philips TUV G13 36 W), providing a maximum output at 254 nm, is used to simulate the atmospheric chemical process of OH + 1,3,5-TMB in lab. A home-made vacuum ultraviolet (VUV) photoionization aerosol mass spectrometer (VUV-AMS) is installed to probe and analyze the particle-phase products inside the Teflon bag. A commercial scanning mobility particle sizer (SMPS, TSI 3936, USA) is used to measure the size distribution of the particles. The quantum chemical method using the Gaussian 16 program is adopted to calculate the parameters of the reactants, intermediates and transition states. The configuration of the VUV photoionization aerosol mass spectrometer and the theoretical methods are described in detail in Ref. [1]

### 2.3 Photoionization mass spectra of the particle-phase products

Raw data of the mass spectra of the particle-phase products from the OH-initiated oxidation reactions of 1,3,5-TMB and deuterated 1,3,5-TMB-d12 (C_9_D_12_) measured by using the VUV photoionization aerosol mass spectrometer can be seen in the Supplementary data. A great deal of peaks can be observed in the mass spectrum and most of them have been assigned (see Table 1). The mass spectrometry data have been deposited to the ProteomeXchange Consortium via the PRIDE (Refs. [2,3]) partner repository with the dataset identifier PXD030839.

### 2.4 The assignments of the main products

The structures of the particle-phase products from the OH-initiated oxidation of 1,3,5-TMB under NO_X_-free conditions are identified. The main peaks in the VUV photoionization mass spectra have been assigned and listed in Table 1, with the aid of the literature results (Refs. [4,5]). For example, the products of the O_2_-bridged bicyclic alcohol (m/z = 186) and carbonyl (m/z = 184), the peroxide (m/z = 202) and the trioxide (m/z = 218) products are clearly observed and identified in the particle-phase. In addition, the ring retaining products of 3,5-dimethyl-phenyl-hydroperoxide (m/z = 138), 3,5-dimethylbenzoic acid (m/z = 150), (3,5-dimethyl-phenyl)-methyl-hydroperoxide (m/z = 152), and an array of low mass oxygenated compounds such as acetone, acetic acid and methyl glyoxal are also observed.

### 2.5 Geometric structures of the main species in the reaction between the BPR radical and HO_2_

The optimized structures of the reactants, reaction intermediates and transition states involved in the reaction between the O_2_-bridged bicyclic peroxy radical (BPR) and HO_2_ on the singlet potential energy surfaces are shown in Fig. 1. The geometric structures were calculated at the M06-2X/MG3S level of theory by using the Gaussian 16 program. The M06-2X functional are hybrid meta density functional theory (DFT) method which has been widely utilized for applications in chemistry (Refs. [6,7]). The selected bond distances such as the hydrogen bond have been given in angstroms.

### 2.6 Formation mechanism of the deuterated bicyclic oxygenated compounds

The reaction routes of the deuterated 1,3,5-TMB (C_9_D_12_) with OH radical under NOx free conditions are presented in Fig. 2. Similar to the reaction of 1,3,5-TMB with OH, the reaction occurs mainly via the OH addition to form the OH-C_9_D_12_ adduct. In the presence of oxygen, the nascent OH-C_9_D_12_ adduct can react with oxygen to generate the OH-C_9_D_12_-O_2_ peroxy radical, which will produce the deuterated bicyclic peroxy radical after subsequent isomerization, reaction with oxygen again and cyclization. Under NOx free condition, reactions of the deuterated bicyclic peroxy radical with the HO_2_ radical, other peroxy radicals, and OH radical play a major role. These reactions will produce the deuterated O_2_-bridged bicyclic alcohol and carbonyl, the peroxide and the trioxide products. In the self-reaction of the deuterated bicyclic peroxy radical, the deuterium-shift results in the products formation with an odd mass number (m/z = 199 and 195), which are observed in the mass spectrum.

## Ethics Statement

The manuscript adheres to ethics in publishing standards.

Appendix A. Supplementary data

*Supplementary data to this article can be found online.* Raw data of the mass spectra of the particle-phase products from the OH-initiated oxidation reactions of 1,3,5-TMB and deuterated 1,3,5-TMB-d12 (C_9_D_12_).

## CRediT authorship contribution statement

**Xiaoxiao Lin:** Investigation, Data curation, Writing – original draft. **Xiaofeng Tang:** Conceptualization, Methodology, Writing – review & editing, Visualization. **Zuoying Wen:** Investigation. **Bo Long:** Investigation, Resources. **Christa Fittschen:** Investigation. **Xuejun Gu:** Investigation. **Yang Zhang:** Investigation. **Weijun Zhang:** Investigation.

## Declaration of Competing Interest

The authors declare that they have no known competing financial interests or personal relationships that could have appeared to influence the work reported in this paper.

## Data Availability

Data of chemical composition of the particles from OH-initiated oxidation of 1,3,5-trimethylbenzene (Original data) (Earth/Chem).

## Appendix Supplementary Materials

Image, application 1
